# Response of the mouse lung transcriptome to welding fume: effects of stainless and mild steel fumes on lung gene expression in A/J and C57BL/6J mice

**DOI:** 10.1186/1465-9921-11-70

**Published:** 2010-06-03

**Authors:** Patti C Zeidler-Erdely, Michael L Kashon, Shengqiao Li, James M Antonini

**Affiliations:** 1 Health Effects Laboratory Division, Pathology and Physiology Research Branch, National Institute for Occupational Safety and Health, Morgantown, 26505, USA; 2 Health Effects Laboratory Division, Biostatistics and Epidemiology Branch, National Institute for Occupational Safety and Health, Morgantown, 26505, USA

## Abstract

**Background:**

Debate exists as to whether welding fume is carcinogenic, but epidemiological evidence suggests that welders are an at risk population for the development of lung cancer. Recently, we found that exposure to welding fume caused an acutely greater and prolonged lung inflammatory response in lung tumor susceptible A/J versus resistant C57BL/6J (B6) mice and a trend for increased tumor incidence after stainless steel (SS) fume exposure. Here, our objective was to examine potential strain-dependent differences in the regulation and resolution of the lung inflammatory response induced by carcinogenic (Cr and Ni abundant) or non-carcinogenic (iron abundant) metal-containing welding fumes at the transcriptome level.

**Methods:**

Mice were exposed four times by pharyngeal aspiration to 5 mg/kg iron abundant gas metal arc-mild steel (GMA-MS), Cr and Ni abundant GMA-SS fume or vehicle and were euthanized 4 and 16 weeks after the last exposure. Whole lung microarray using Illumina Mouse Ref-8 expression beadchips was done.

**Results:**

Overall, we found that tumor susceptibility was associated with a more marked transcriptional response to both GMA-MS and -SS welding fumes. Also, Ingenuity Pathway Analysis revealed that gene regulation and expression in the top molecular networks differed between the strains at both time points post-exposure. Interestingly, a common finding between the strains was that GMA-MS fume exposure altered behavioral gene networks. In contrast, GMA-SS fume exposure chronically upregulated chemotactic and immunomodulatory genes such as *CCL3*, *CCL4*, *CXCL2*, and *MMP12 *in the A/J strain. In the GMA-SS-exposed B6 mouse, genes that initially downregulated cellular movement, hematological system development/function and immune response were involved at both time points post-exposure. However, at 16 weeks, a transcriptional switch to an upregulation for neutrophil chemotactic genes was found and included genes such as *S100A8*, *S100A9 *and *MMP9*.

**Conclusions:**

Collectively, our results demonstrate that lung tumor susceptibility may predispose the A/J strain to a prolonged dysregulation of immunomodulatory genes, thereby delaying the recovery from welding fume-induced lung inflammation. Additionally, our results provide unique insight into strain- and welding fume-dependent genetic factors involved in the lung response to welding fume.

## Background

The harmful health effects of welding are well documented and epidemiological evidence generally supports the hypothesis that exposure to welding fume increases lung cancer risk, but confounders such as asbestos exposure and smoking obscure these findings [1-4]. Debate also exists over which type of welding may pose the greater risk. Welding processes that use stainless steel (SS) wire produce fumes that contain carcinogenic metals such as chromium and nickel. Welding fume from mild steel (MS) wire, the type most used in the workplace, primarily consists of iron with a lesser amount of manganese, but no chromium or nickel. Interestingly, fumes from both MS and SS welding have been shown to increase lung cancer risk in this worker population [5,6].

The International Agency for Research on Cancer has deemed welding fume a group 2B agent, defined as a mixture "possibly carcinogenic" to humans [7]. However, this categorization of welding fume carcinogenicity was based on limited evidence in humans and virtually no animal data. For these reasons, we initiated a series of studies to ultimately determine the carcinogenic potential of welding fume in an animal model.

A/J mice are genetically predisposed to spontaneous and/or chemically-induced lung tumors while C57BL/6J (B6) mice are essentially resistant [8]. In a recent study, we found that exposure by pharyngeal aspiration to welding fume caused lung inflammation (polymorphonuclear leukocyte [PMN] influx) and increased lung cytotoxicity, permeability and cytokine production (IL-6, TNF-α and MCP-1) in the bronchoalveolar lavage (BAL) of both A/J and B6 mice. The A/J strain, however, exhibited a significantly greater lung response magnitude and an attenuated resolution of the response compared to the resistant B6 strain. We also found that the SS fumes, particularly those of an insoluble type derived from gas metal arc (GMA) welding, were more biopersistent than the GMA-MS fumes, provoked a mild chronic inflammation in the A/J lung and tended to cause the greatest, overall, lung toxicity. Furthermore, we observed a trend for an increased lung tumor incidence in the GMA-SS welding fume-exposed A/J mice, which, when considered in conjunction with our other findings, suggested that a chronic lung response to GMA-SS welding fume may enhance tumorigenesis in the A/J model [9]. In this study, we rationalized that these strain-dependent differences would provide a unique backdrop to examine underlying inflammatory and possibly tumorigenic mechanisms associated with welding fume exposure at the transcriptome level. Although considerable information has been gleaned by exploring the lung toxicity of welding fume *in vivo*, specific knowledge of the genes expressed in the context of welding fume-induced lung toxicity is only beginning to emerge [10-13].

Recent technical advances in functional genomics have led to the global and simultaneous analysis of gene expression in cells or tissues at the level of transcription. Microarray offers the opportunity to comprehensively probe alterations in the genome within experimentally manipulated samples. Utilizing software applications such as Ingenuity Pathways Analysis (IPA) provides the vast knowledge base needed to interpret large microarray datasets and generate understandable molecular and biological networks based on key findings. Thus, our objective was to characterize lung gene expression in two genetically distinct mouse strains, A/J and B6, exposed to either MS or SS welding fume in a comprehensive manner using microarray and IPA. We hypothesized that differences would exist--transcriptionally--between these strains and that the A/J may display a tendency toward continued activation of inflammatory genes or early activation of oncogenes compared to the B6 strain. Collectively, our results demonstrate that lung tumor susceptibility may predispose the A/J strain to a prolonged dysregulation of immunomodulatory genes, thereby delaying the recovery from welding fume-induced lung inflammation. Additionally, our results provide unique insight into strain- and welding fume-dependent genetic factors involved in the lung response to welding fume.

## Methods

### Animals

Male A/J and B6 mice, 4 weeks of age were purchased from Jackson Laboratories (Bar Harbor, ME) and housed in an AAALAC-accredited, specific pathogen-free, environmentally controlled facility. All mice were free of endogenous viral pathogens, parasites, mycoplasmas, Helicobacter and CAR Bacillus. Mice were individually housed in ventilated cages and provided HEPA-filtered air under a controlled light cycle (12 hour light/12 hour dark) at a standard temperature (22-24°C) and 30-70% relative humidity. Animals were acclimated to the animal facility for a minimum of 1 week and allowed access to a conventional diet (6% Irradiated NIH-31 Diet, Harlan Teklad, Madison, WI) and tap water *ad libitum*. All procedures were performed using protocols approved by the National Institute for Occupational Safety and Health Institutional Animal Care and Use Committee.

### Welding fume collection and characterization

The welding fumes used in this study were provided by Lincoln Electric Co. (Cleveland, OH). The collection and characterization of these fumes were previously described [14]. Briefly, the fumes were generated in a cubical open-front fume chamber (volume = 1m^3^) by a skilled welder, using a manual or automatic technique appropriate for the electrode and then collected on a 0.2 μm filter. The samples were generated by gas metal arc welding (with argon and CO_2 _shielding gases) using a mild steel electrode or a stainless steel electrode. The metal constituents, solubility/insolubility ratio and pH of each welding fume sample were previously reported [9]. Briefly, seven different metals (Cr, Cu, Fe, Mn, Ni, Ti and V) commonly found in welding fumes were measured using inductively coupled argon plasma atomic emission spectroscopy. GMA-SS welding fume consisted of the following metals (weight %): Fe (53.1), Cr (18.6), Mn (23.2), Ni (4.85) with trace amounts of Cu. GMA-MS fume consisted of 85.9% Fe and 14.6% Mn with trace amounts of Cr (0.07), Cu (0.41), Ni (0.01) and Ti (0.02). The soluble/insoluble ratios of the GMA-MS and -SS fumes were 0.020 and 0.006, respectively.

### Welding fume preparation

Each welding fume was weighed and suspended in sterile Ca^+2 ^and Mg^+2^-free PBS in a 50 ml sterile conical tube. Count mean diameters were 1.22 and 1.38 μm for the GMA-MS and GMA-SS fumes, respectively, as determined by electron microscopy [14]. Following the initial preparation, the fume samples were vortexed then sonicated for 1 minute using a Sonifier 450 Cell Disruptor (Branson Ultrasonics, Danbury, CT). Prior to dosing, the samples were vortexed then sonicated for 15 seconds and vortexed immediately before each mouse exposure. For each experimental time point, fresh welding fume suspensions were made and the same preparation was used to expose both strains of mice.

### Mouse pharyngeal aspiration exposure

Age and weight-matched mice were exposed to GMA-MS, GMA-SS or sterile Ca^+2 ^and Mg^+2^-free PBS vehicle (sham) by pharyngeal aspiration as previously described [15]. Briefly, each mouse was placed in a glass jar with a gauze pad moistened with isoflurane (Abbott Laboratories, North Chicago, IL) until slowed breathing was observed. The mouse was then suspended, by its top incisors, on a slanted board in a dorsal recumbent position. The tongue was extended with forceps and the solution was pipetted to the oropharynx. The tongue was held extended until the solution was aspirated into the lung and the mouse resumed a regular breathing pattern. When performed properly, this technique allows minimal sample loss to the digestive tract. The mouse was then returned to its cage to recover, typically 10-15 seconds.

In this study, mice were exposed over a 10 day period to 4 bolus doses of test material in lieu of a single bolus dose. This regime achieved an accumulation of particles in the lung over time, which may be more representative of an occupational exposure. Mice were exposed 4 times (once every 3 days) to 85 μg (~5 mg/kg) of GMA-MS or GMA-SS welding fume. The cumulative fume lung burden was derived from our previous pharyngeal aspiration experiment in the A/J mouse and is equivalent to ~196 days of exposure in a 75 kg welder working an 8 hour shift [16]. A 25 μl aspiration volume was used and shams were administered an equal volume of PBS. Mice were euthanized 4 and 16 weeks after the fourth exposure. We chose to examine 4 weeks post-exposure based on our previous data that showed the lung response to welding fume was resolving in both mouse strains by this time [9]. We also evaluated 16 weeks post-exposure for chronic lung transcriptional alterations to welding fume. At 16 weeks, there was no evidence of any ongoing histopathologic response to either welding fume in the A/J lung, although both welding fumes were still present in significant amounts (unpublished observation).

### Body weight determination

Mice were weighed after the 1 week acclimation period, throughout the dosing and again at 4 and 16 weeks. All groups gained weight throughout the study and no treatment effects were observed.

### Whole lung RNA isolation

Mice were anesthetized with an intraperitoneal overdose of Sleepaway (26% sodium pentobarbital, 7.8% isopropyl alcohol and 20.7% propylene glycol, Fort Dodge Animal Health, Fort Dodge, IA) then weighed. Once the mouse was unresponsive to a toe pinch, the abdomen was opened and the vena cava was cut to exsanguinate the mouse. Whole lungs were removed from sham and welding fume-exposed mice then snap frozen in liquid nitrogen and stored at -80°C for RNA isolation. RNA was isolated from whole lung homogenates using the TRIzol (Invitrogen, Carlsbad, CA) method and then cleaned according to the manufacturer's instructions using a RNeasy Mini Kit (Qiagen, Valencia, CA). A 2 μl aliquot of each RNA sample was quantified using a NanoDrop ND-1000 spectrophotometer (NanoDrop Technologies, Inc., Wilmington, DE) and quality was assessed on the Agilent 2100 Bioanalyzer (Agilent Technologies, Palo Alto, CA).

### MouseRef-8 v1.1 Illumina BeadChips

Labeled cRNA, from an input RNA of 375 ng, was prepared according to the manufacture's protocol, using the Illumina TotalPrep RNA Amplification Kit (Applied Biosystems Inc., Foster City, CA, Catalog #AMIL1791) for hybridization to the arrays. The labeled cRNA samples were then assessed for quality and quantity. To ensure consistency for the array hybridization, all cRNA samples for each time point were quantified at the same time. The MouseRef-8 v1.1 beadchip contains > 24,000 well annotated RefSeq transcripts and allows 8 samples to be interrogated in parallel. To minimize array to array variability, a cRNA sample from each of the sham, GMA-MS and GMA-SS groups from both mouse strains was hybridized to each of the beadchips (n = 4/group/strain) according to the manufacturer's protocol. After a 20 hour hybridization period at 58°C, the beadchips were scanned using an Illumina BeadStation 500 G - BeadArray Reader (Illumina, Inc., San Diego, CA). The data discussed in this publication were deposited in NCBI's Gene Expression Omnibus (GEO) [17]. Data are accessible through GEO Series accession number GSE20174 http://www.ncbi.nlm.nih.gov/geo/.

### Statistics and data analysis strategy for Illumina beadchips

Metrics files from the bead scanner were checked to ensure that all samples fluoresced at comparable levels before importing samples into Beadstudio (Framework version 3.0.19.0) Gene Expression module v.3.0.14. Reference, hybridization control, stringency and negative control genes were checked for proper chip detection. Beadarray expression data were then exported with mean fluorescent intensity across like beads and bead variance estimates into flat files for subsequent analysis.

Illumina BeadArray expression data were analyzed in Bioconductor using the 'lumi' and 'limma' packages. Bioconductor is a project for the analysis and comprehension of genomic data and operates in R, a statistical computing environment [18]. The 'lumi' Bioconductor package was specifically developed to process Illumina microarrays and covers data input, quality control, variance stabilization, normalization and gene annotation [19]. Background correction utilized the method known as force positive to force all expression values to be positive by adding an offset (minus minimum values plus 1). This background correction precedes the variance stabilizing transformation (VST) method which takes advantage of the technical replicates available on an Illumina microarray. Data normalization proceeds using the robust spline normalization algorithm, which combines the features of quantile and loess normalization [19]. Prior to subsequent analyses including differential expression analysis, unexpressed genes were filtered out.

Normalized data were then analyzed using the 'limma' package in R. The 'limma' package is designed to fit specific linear models for microarray data., generates group means of expression, p-values are calculated (including adjusted p-values for multiple tests) and log fold-changes which are converted to standard fold changes. These lists of genes and their associated statistics are utilized as input for subsequent bioinformatic analysis.

### Hierarchical clustering

Heat maps for the 4 and 16 week time points were generated using the gplots package in R with the default settings of Euclidean distance and complete linkage for the construction of the dendrograms.

### Molecular Network Analysis using Ingenuity Pathways Analysis (IPA)

Data were analyzed using Ingenuity Pathways Analysis (IPA version 6.3) (Ingenuity Systems^®^, http://www.ingenuity.com). Whole datasets containing gene identifiers and corresponding expression values were uploaded into the application and a core analysis was done. Each identifier was mapped to its corresponding gene object in the Ingenuity knowledge base. A fold change cutoff of 1.3 was set to identify genes whose expression was significantly differentially regulated. These genes, called focus genes, were overlaid onto a global molecular network developed from information contained in the Ingenuity knowledge base. Networks of these focus genes were then algorithmically generated based on their connectivity. For simplicity, the most significant network (highest network score or lowest p-value) generated by IPA which incorporated the greatest number of the focus genes is presented. Network scores are calculated using Fisher's exact test and is equal to the -log_10 _(p-value).

The Biological Functional Analysis identified the biological functions and/or diseases that were most significant to the data set. Genes from the dataset that met the fold change cutoff of 1.3 and were associated with biological functions and/or diseases in the Ingenuity knowledge base were considered for the analysis. Fischer's exact test was used to calculate a p-value determining the probability that each biological function and/or disease assigned to that data set is due to chance alone.

### Confirmation of microarray data by RT-qPCR

A gene subset from the 4 week time point differentially expressed in the A/J strain by microarray was confirmed using the following Pre-designed Assays-on-Demand™ TaqMan^® ^probes and primers from Applied Biosystems: complement factor B (*CFB*) [Mm00433909_m1], lipocalin 2 (*LCN2*) [Mm01324472_g1], matrix metalloproteinase 12 (*MMP12*) [Mm00500554_m1], osteopontin (*SPP1*) [Mm00436767_m1]. One μg of total RNA was reverse-transcribed using random hexamers (Applied Biosystems, Foster City, CA) and Superscript II (Invitrogen, Carlsbad, CA). Five μl of cDNA (in duplicates for each gene) was then used for gene expression determination using the Applied Biosystems 7900 HT (Foster City, CA). The ribosomal subunit 18 S was used as the reference gene (Hs99999901_s1, Applied Biosystems). Relative gene expression was calculated using the comparative threshold method (2-ΔΔCt) [20]. All genes were validated in both GMA-MS and -SS exposed lungs except *SPP1*, which was only confirmed in the GMA-SS A/J lung tissue. The same lung RNA samples were used for both RT-qPCR and microarray gene expression analysis. Data were analyzed by one-way analysis of variance (ANOVA) generating a least squares means table by Student's t-test using JMP^® ^Statistical Discovery Software.

## Results and Discussion

### Hierarchical clustering

**Figure 1:**
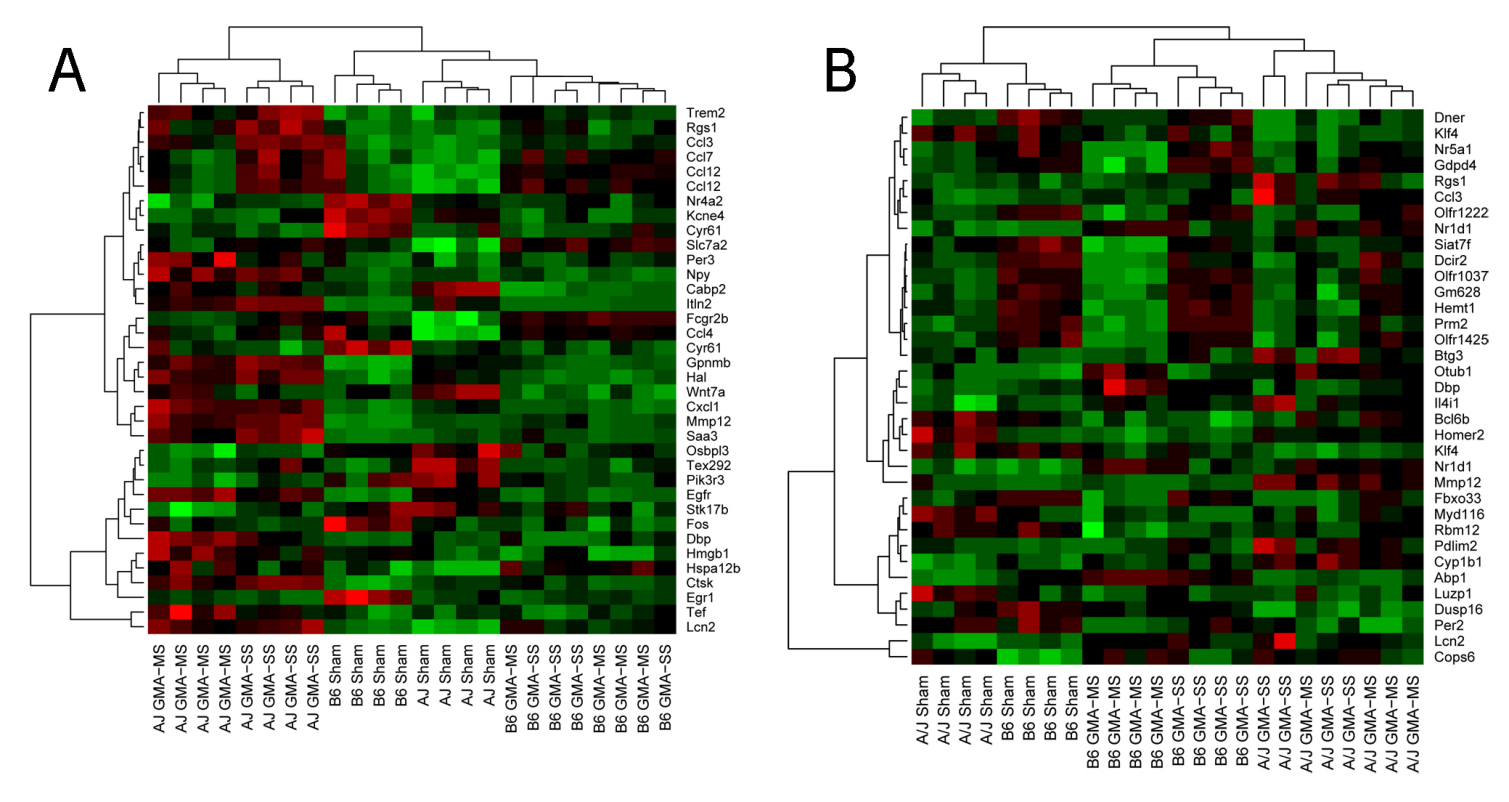
**Hierarchical clustering of differentially expressed genes in GMA welding fume-exposed A/J and B6 mice**. Hierarchical clustering analysis of differentially expressed genes in the lungs of A/J and B6 mice exposed to GMA-MS or GMA-SS welding fume or PBS (sham) at 4 (panel A) and 16 weeks (panel B) post-exposure after FDR p-value adjustment (p < 0.05, n = 4/group). The range of gene expression values are represented as the color scheme green-black-red which indicates low-moderate-high gene expression compared to the corresponding sham. Note: Two different Illumina probe sequences on the MouseRef-8 v1.1 beadchip were present for *CCL12*, *KLF4*, *NR1D1*; therefore, these genes appear twice on the heatmap.

Shown in figure 1 are the heatmaps of differentially expressed genes in the lungs of A/J and B6 mice exposed to GMA-MS, GMA-SS welding fume or vehicle at 4 (panel A) and 16 weeks (panel B) post-exposure. Comparisons were made to the corresponding control mouse strain. Overall, at 4 and 16 weeks, expression patterns of the analyzed genes were more similar within exposure groups rather than between exposure groups. This indicates that a good consistency across samples was found on the individual arrays. Using our statistical criteria, 36 annotated genes resulted in 5 distinct subclusters among the A/J and B6 welding fume-exposed and control groups at 4 weeks post-exposure. The subclusters intermixed in the GMA-MS or -SS exposed B6 representing similar gene expression patterns within this strain to these welding fumes; this was in contrast to the A/J strain (see panel A).

By 16 weeks post-exposure, 35 annotated genes resulted in 5 distinct subclusters among the A/J and B6 welding fume-exposed and control groups. In contrast to our 4 week analysis, the subclusters intermixed in the GMA-MS or -SS exposed A/J, representing similar gene expression patterns within this strain to these welding fumes by 16 weeks (see panel B).

### Gene activation 4 and 16 weeks post-exposure to welding fume

**Figure 2:**
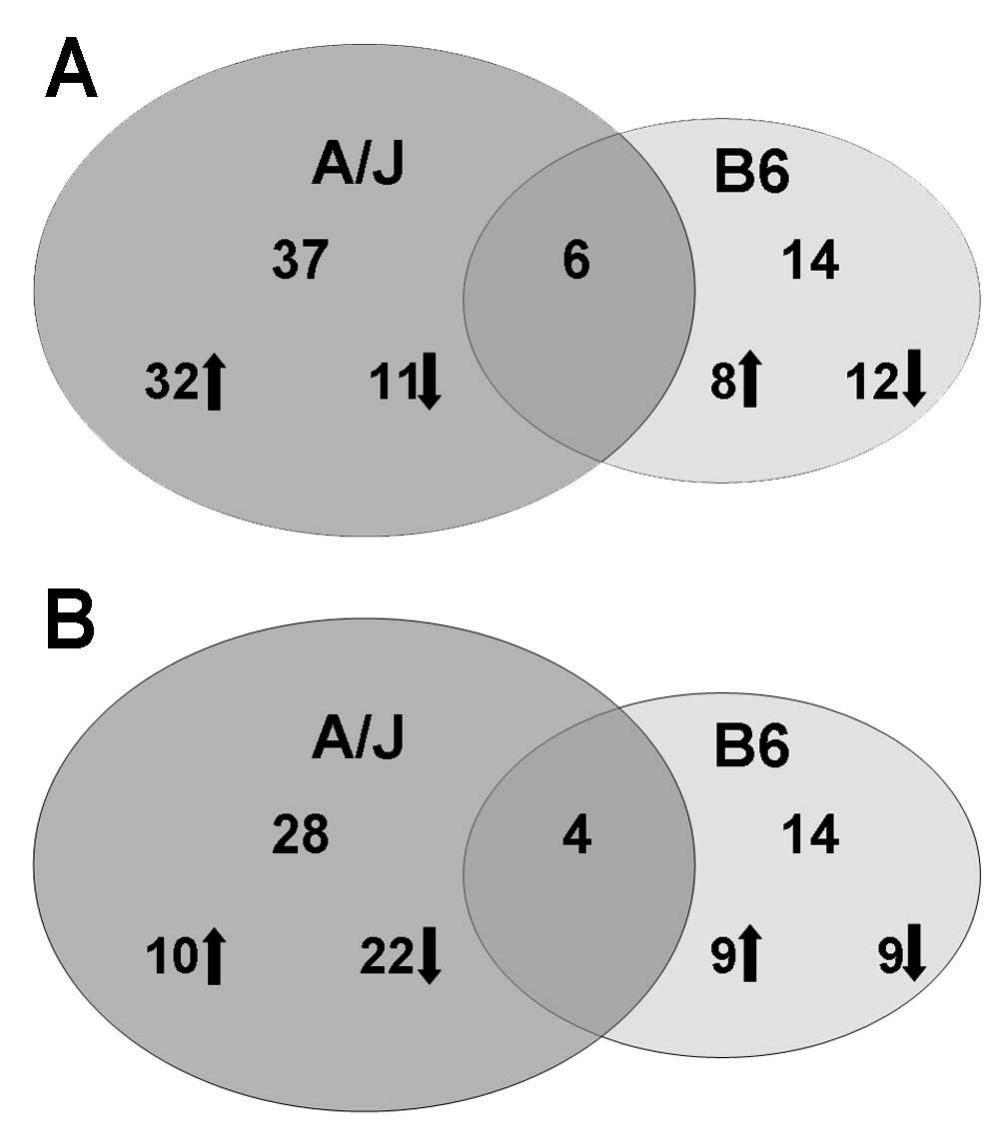
**Differential gene regulation after GMA-MS welding fume exposure in A/J and B6 mice**. Comparison of the number of differentially expressed genes in the lungs of A/J and B6 mice exposed to GMA-MS welding fume at 4 (panel A) and 16 weeks (panel B) post-exposure. The number of genes upregulated (↑) and downregulated (↓) are shown for each strain. At 4 weeks, GMA-MS welding fume exposure induced 6 common genes between the strains: *CH25H*, chromosome 10 open reading frame 10 (*C10ORF10*), *KLF2*, *KLF4*, macrophage receptor with collagenous structure (*MARCO*) and natriuretic peptide receptor C/guanylate cyclase C (*NPR3*). At 16 weeks, 4 common genes were differentially expressed: *DNAJB1*, *LCN2*, *NR1D1 *and *PER2*. Whole datasets for each strain were uploaded into IPA then analyzed with the cutoff criteria of ≥1.3 fold change and p < 0.05 versus corresponding sham.

**Figure 3:**
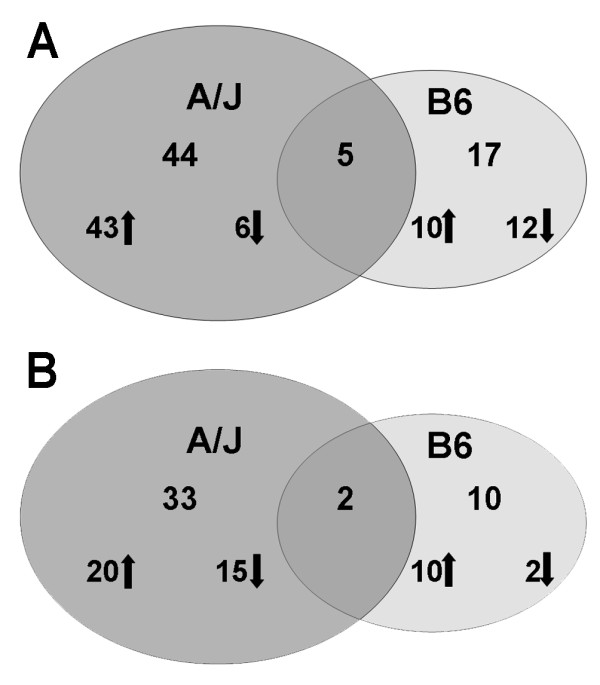
**Differential gene regulation after GMA-SS welding fume exposure in A/J and B6 mice**. Comparison of the number of differentially expressed genes in the lungs of A/J and B6 mice exposed to GMA-SS welding fume at 4 (panel A) and 16 weeks (panel B) post-exposure. The number of genes upregulated (↑) and downregulated (↓) are shown for each strain. GMA-SS fume exposure induced 5 common genes at 4 weeks post-exposure: cathespin K (*CTSK*), *HSPH1*, *MMP12*, *PER2 *and solute carrier family 26, member 4 (*SLC26A4*). Only 2 common genes were induced at 16 weeks post-exposure: *DNAJB1 *and *NR1D1*. Whole datasets for each strain were uploaded into IPA then analyzed with the cutoff criteria of ≥1.3 fold change and p < 0.05 versus corresponding sham.

Based on our selected analysis criteria, at 4 weeks after GMA-MS fume exposure the A/J strain had an overall upregulation in gene transcription compared with the B6. Nearly three quarters (32 out of 43) of the genes in the A/J lung were upregulated versus only 40% (8 out of 20) in the B6 strain at this time point (Figure 2, panel A). By 16 weeks post-exposure, the A/J exhibited an overall downregulation in gene transcription after GMA-MS compared with the B6, 69% (22 out of 32) versus 50% (9 out of 18), respectively (Figure 2, panel B). Similarly, with GMA-SS exposure, 88% (43 out of 49) of the genes were upregulated in the A/J, whereas 45% (10 out of 22) in the B6 were upregulated (Figure 3, panel A). At 16 weeks post-exposure to GMA-SS, the number of differentially expressed genes in the A/J was 35 versus 12 in the B6 strain. Of the genes analyzed, 83% (10 out of 12) in the B6 and 57% (20 out of 35) in the A/J strain were upregulated (Figure 3, panel B). These data collectively show a more marked response in the A/J at both time points and with both welding fumes.

### 4 weeks post-exposure to GMA-MS: IPA analysis

**Figure 4:**
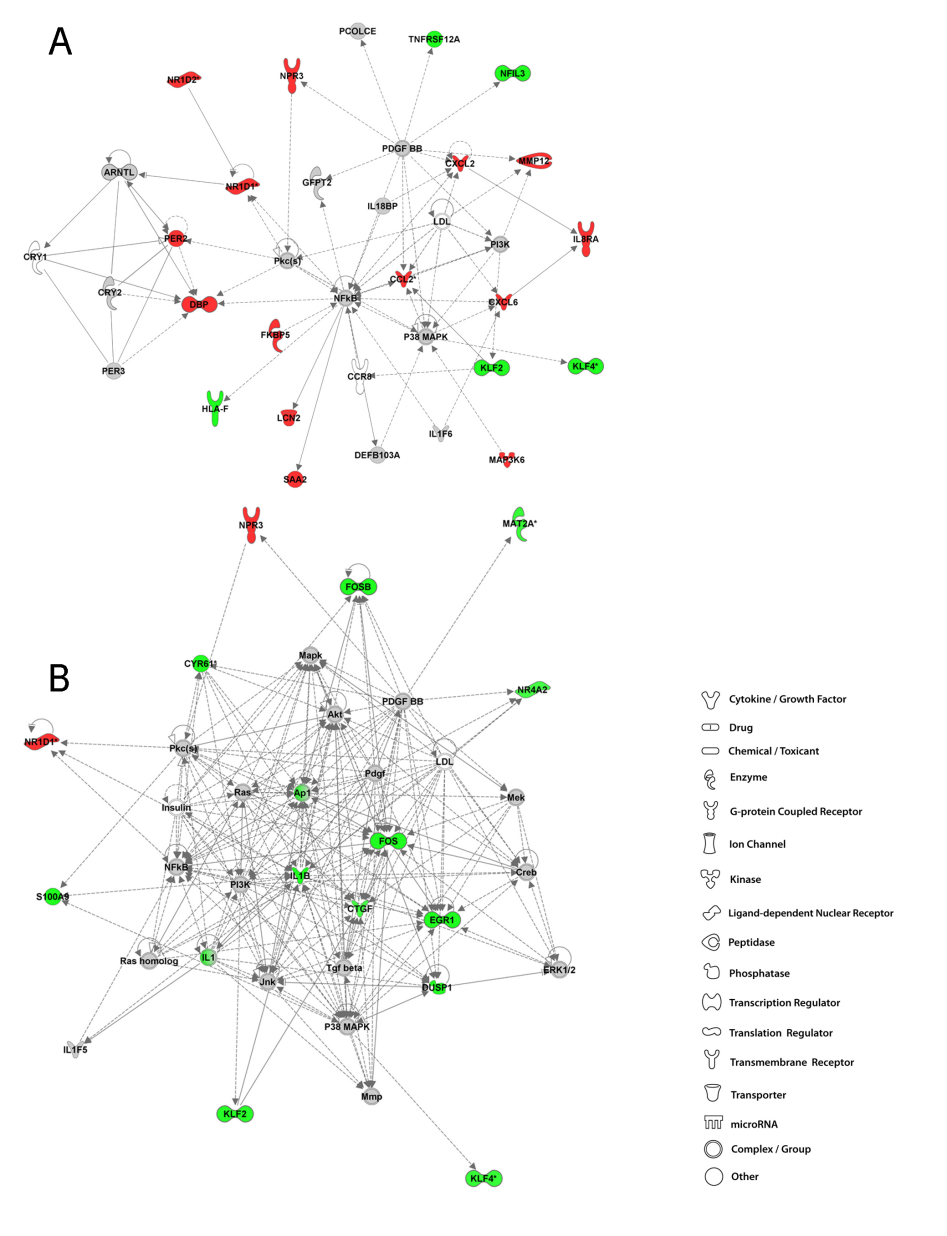
**Top molecular networks 4 weeks after GMA-MS welding fume exposure in A/J and B6 mice**. Gene network analysis by IPA of differentially expressed focus genes 4 weeks after exposure to GMA-MS welding fume in A/J (panel A) and B6 (panel B) mice. Whole datasets for each strain were uploaded into IPA then analyzed with the cutoff criteria of ≥1.3 fold change and p < 0.05 versus corresponding sham. Only the highest scoring or most significant network is shown for each group. Intensity of the red (upregulated) or green (downregulated) color indicates level of gene expression. The white color indicates a predicted molecule incorporated from the Ingenuity knowledge base. Gray represents a molecule present in the dataset, but one that did not meet the specified cutoff criteria.

IPA analysis is unbiased and independent of the study design. The networks generated from the input of transcriptional data yields networks based on the known functions and interconnectivity of the affected genes. Therefore, network titles refer to the primary functions of the gene pathways. Network analysis shows upregulated (intensity of red) and downregulated (intensity of green) molecules with the remaining pathway molecules incorporated by IPA. Molecules that were not user specified, but incorporated into the network through relationships with other molecules are white and those that were neither up nor down regulated or did not meet the defined cutoff criteria are gray. The top network in the A/J lung at 4 weeks post-exposure to GMA-MS was behavior, nervous system development and function and gene expression which incorporated 19 focus genes out of 43 network eligible genes (Figure 4, panel A). Genes commonly associated with an inflammatory lung response were altered including kruppel-like factor 2 (lung) [*KLF2*], chemokine (C-C motif) ligand 2 (*CCL2*), chemokine (C-C motif) receptor 8 *(CCR8) *and nuclear factor interleukin 3 regulated (*NFIL3*). Predicted involvement, by IPA, of other molecules involved in this network were the nuclear factor-kappa B (NFκB) complex, platelet-derived growth factor BB (PDGF BB), p38 mitogen- activated protein kinase (MAPK) and the phosphoinositide 3-kinase (PI3K) complex. Lending to the title of this network was the alteration of genes under the higher level function of behavior and nervous system development and function. These genes including D site of albumin promoter (albumin D-box) binding protein (*DBP*), period circadian protein homolog 2 (*PER2*), and nuclear receptor subfamily 1, group D member 1 and 2 (*NR1D1 *and *NR1D2) *are important in circadian rhythm signaling, but also may have functional roles in lung pathobiology and/or lung tumorigenesis [21,22].

The response in the B6 GMA-MS-exposed lung involved a significant transcriptional downregulation and included genes involved in the higher level disease and disorder category of cancer, functional subcategory apoptosis (Figure 4, panel B). These genes included transcriptional regulators early growth response protein 1 (*EGR1*), *KLF2*, *KLF4*, nuclear receptor subfamily 4, group A, member 2 (*NR4A2*) and members of the v-fos FBJ murine osteosarcoma viral oncogene homolog family or *FOS *genes. An important macrophage-derived gene, interleukin 1 beta (*IL1β*), which regulates the acute phase response, was also downregulated at this time point.

### 16 weeks post-exposure to GMA-MS: IPA analysis

**Figure 5:**
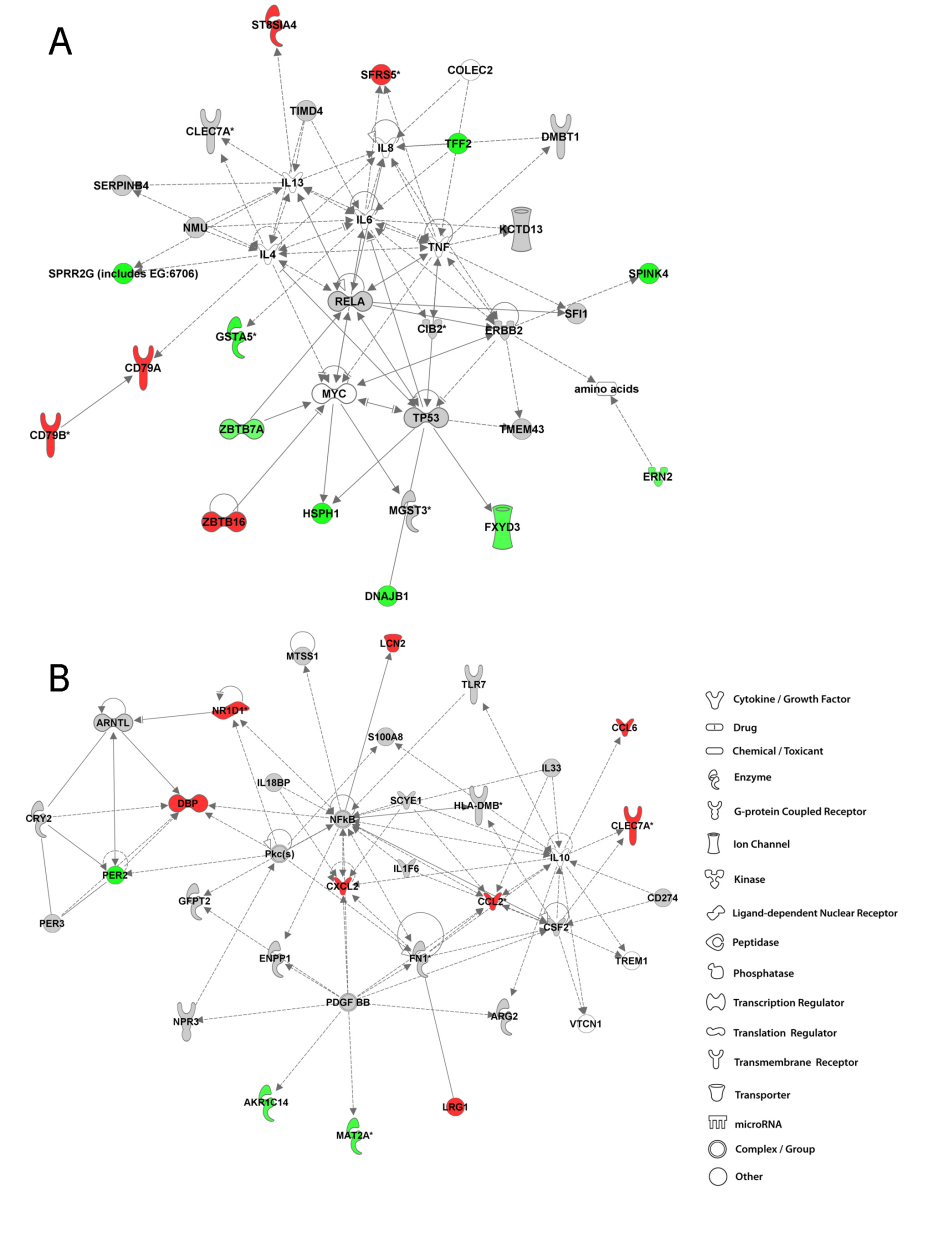
**Top molecular networks 16 weeks after GMA-MS welding fume exposure in A/J and B6 mice**. Gene network analysis by IPA of differentially expressed focus genes 16 weeks after exposure to GMA-MS welding fume in A/J (panel A) and B6 (panel B) mice. Whole datasets for each strain were uploaded into IPA then analyzed with the cutoff criteria of ≥1.3 fold change and p < 0.05 versus corresponding sham. Only the highest scoring or most significant network is shown for each group. Intensity of the red (upregulated) or green (downregulated) color indicates level of gene expression. The white color indicates a predicted molecule incorporated from the Ingenuity knowledge base. Gray represents a molecule present in the dataset, but one that did not meet the specified cutoff criteria.

At the later time point after GMA-MS exposure in the A/J, predicted involvement of oncogenes associated with cell survival pathways included tumor protein 53 (*TP53*) that encodes p53 protein and v-myc myelocytomatosis viral oncogene homolog [avian] (*MYC*) (Figure 5, panel A). Genes both up and downstream from *TP53 *and *MYC *were those with functional roles in cellular stress responses and/or cell death and included DnaJ homolog subfamily B member 1(*DNAJB1*), heat shock protein 105 kDa *(HSPH1) *and zinc finger and BTB domain containing 16 (*ZBTB16) *which was also upregulated at 4 weeks (2.4 fold). Interestingly, continued involvement of circadian rhythm signaling genes (second highest rated network) was also found. At 16 weeks, predicted molecules included the NFκB family of transcription factors, particularly v-rel reticuloendotheliosis viral oncogene homolog A (avian) or *RELA.*

The response in the B6 GMA-MS-exposed lung at 16 weeks involved 8 genes that were upregulated in the top network including the inflammatory cytokines *CCL2 *and chemokine (C-X-C motif) ligand 2 (*CXCL2) *(Figure 5, panel B). A behavioral gene subset was differentially regulated in the B6 at 16 weeks and this network component was also present in the top network of the A/J strain at 4 weeks post-exposure to GMA-MS fume. A conserved, consistent expression of one of the behavioral genes *NR1D1 *was found. Expression levels were 2.3 and 2.2 fold for *NR1D1 *at 4 and 16 weeks, respectively. NFκB was a predicted molecule at this time point which formed a direct relationship with interleukin 1 (IL-1)-induced inflammatory gene, *LCN2*, or oncogene 24p3.

### Summary of network discovery after GMA-MS welding fume exposure

In our previous study, at 4 weeks after GMA-MS welding fume exposure, minimal but significant lung cytotoxicity and inflammation persisted in the A/J strain, whereas inflammation resolved in the B6 by 7 days [9]. Our lung transcriptome profiling in these mouse strains complements these findings. More specifically, an attenuated downregulation of the transcriptome and a greater number of affected genes in the A/J strain compared to the B6 was found. Some gene networks altered during the early and late resolution phases of the lung response to GMA-MS fume were not as expected. Although, anti-inflammatory signaling was occurring in the B6 at 4 weeks (i.e., downregulation of *IL1β*, *FOS*, S100 calcium binding protein A9 [*S100A9*], etc.) other "later" gene interactions were surprising (Figures 4 and 5). Perhaps the most intriguing finding regarding GMA-MS welding fume exposure was the differential expression of behavioral genes associated with circadian rhythm signaling. Most notably, we found a consistent increased expression of *NR1D1 *in both mouse strains at 4 and 16 weeks post-exposure. Although primarily characterized as a circadian rhythm regulatory gene, *NR1D1 *is implicated as a tumor suppressor gene and may modulate cell proliferation/differentiation and NF-κB pathways, a common hub in the GMA-MS gene networks [23-25]. Consistent with our findings, previous studies also revealed changes in murine lung expression of circadian rhythm genes, including *NR1D1*, following cigarette smoke exposure [21]. These findings suggest that this particular gene subset may be important in the lung response to toxic stimuli.

### 4 weeks post-exposure to GMA-SS: IPA analysis

**Figure 6:**
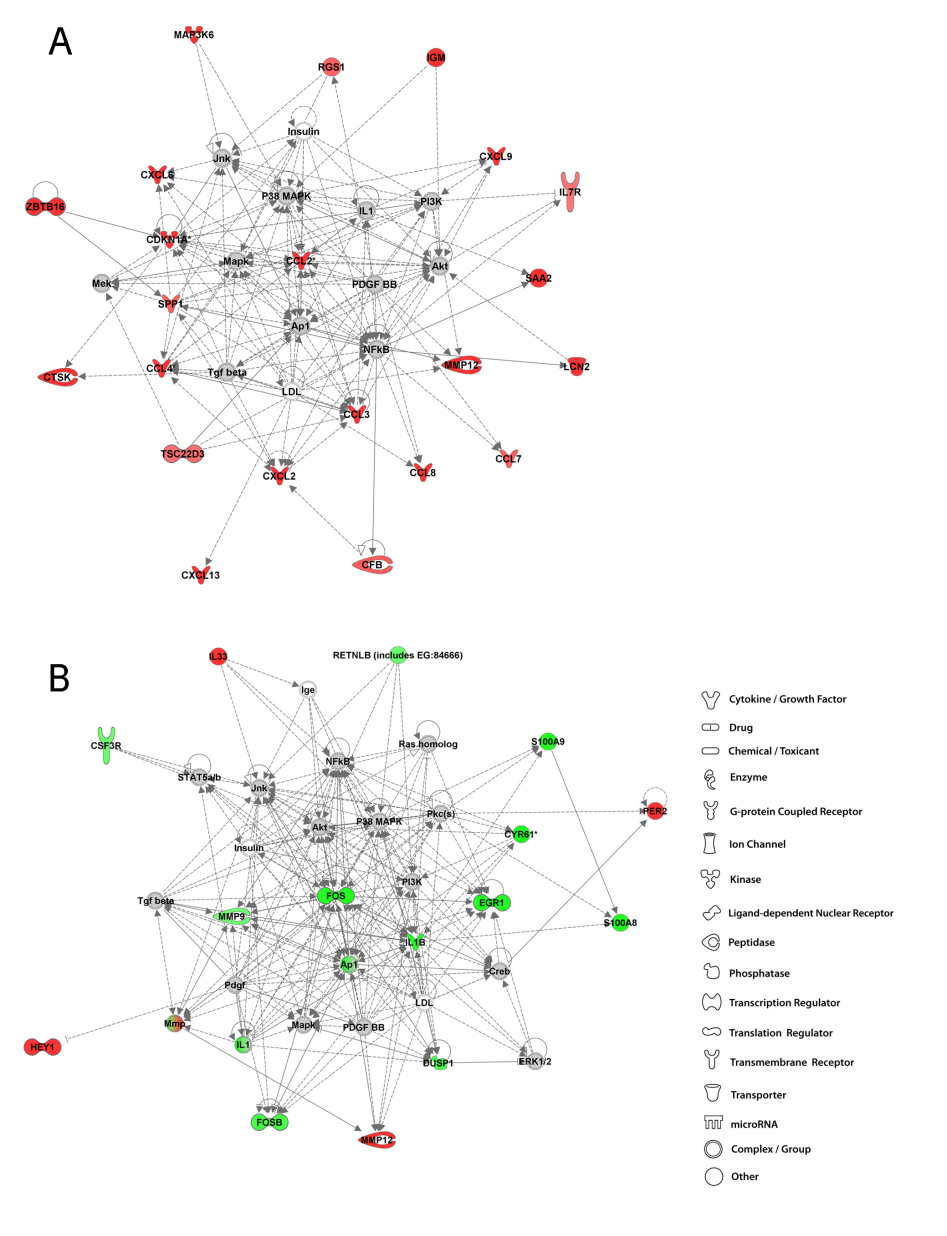
**Top molecular networks 4 weeks after GMA-SS welding fume exposure in A/J and B6 mice**. Gene network analysis by IPA of differentially expressed focus genes 4 weeks after exposure to GMA-SS welding fume in A/J (panel A) and B6 (panel B) mice. Whole datasets for each strain were uploaded into IPA then analyzed with the cutoff criteria of ≥1.3 fold change and p < 0.05 versus corresponding sham. Only the highest scoring or most significant network is shown for each group. Intensity of the red (upregulated) or green (downregulated) color indicates level of gene expression. The white color indicates a predicted molecule incorporated from the Ingenuity knowledge base. Gray represents a molecule present in the dataset, but one that did not meet the specified cutoff criteria.

In the A/J GMA-SS-exposed lung, the top network included 22 significantly upregulated focus molecules such as inflammatory chemokines regulating cell (monocyte, natural killer, and neutrophil) movement such as *CCL2 *and *CCL4 *and *CXCL2 *(Figure 6, panel A). Increased transcriptional activity was also found for genes involved in the higher level disease and disorder category of immunological disease including the acute phase response protein serum amyloid 2 (*SAA2*), *ZBTB16 *and osteopontin. Predicted molecular involvement in this network were the Akt protein family (protein kinase B), the NFκB complex, activator protein-1 (AP-1), p38 MAPK and Mek.

The top network in the B6 GMA-SS-exposed lung consisted primarily of decreased gene expression for dual specificity phosphatase 1 (*DUSP1*), a downregulator of MAPK signaling, transcriptional regulators *EGR1*, *FOS, FOSB*, and pro-inflammatory cytokine *IL1β *(Figure 6, panel B). These gene interactions were also present in the B6 response to GMA-MS welding fume, which suggests similar transcriptional regulation to both MS and SS fumes in this strain at 4 weeks (Figures 4B and 6B). Cellular movement, a top molecular and cellular function associated with GMA-SS in the B6, encompassed an overall downregulation of a gene subset involved in movement of leukocytes, lymphatic system and blood cells; these included colony stimulating factor 3 receptor [granulocyte] (*CSF3R*), *DUSP1*, *IL1β*, *MMP9*, *S100A8 *and *S100A9*.

### 16 weeks post-exposure to GMA-SS: IPA analysis

**Figure 7:**
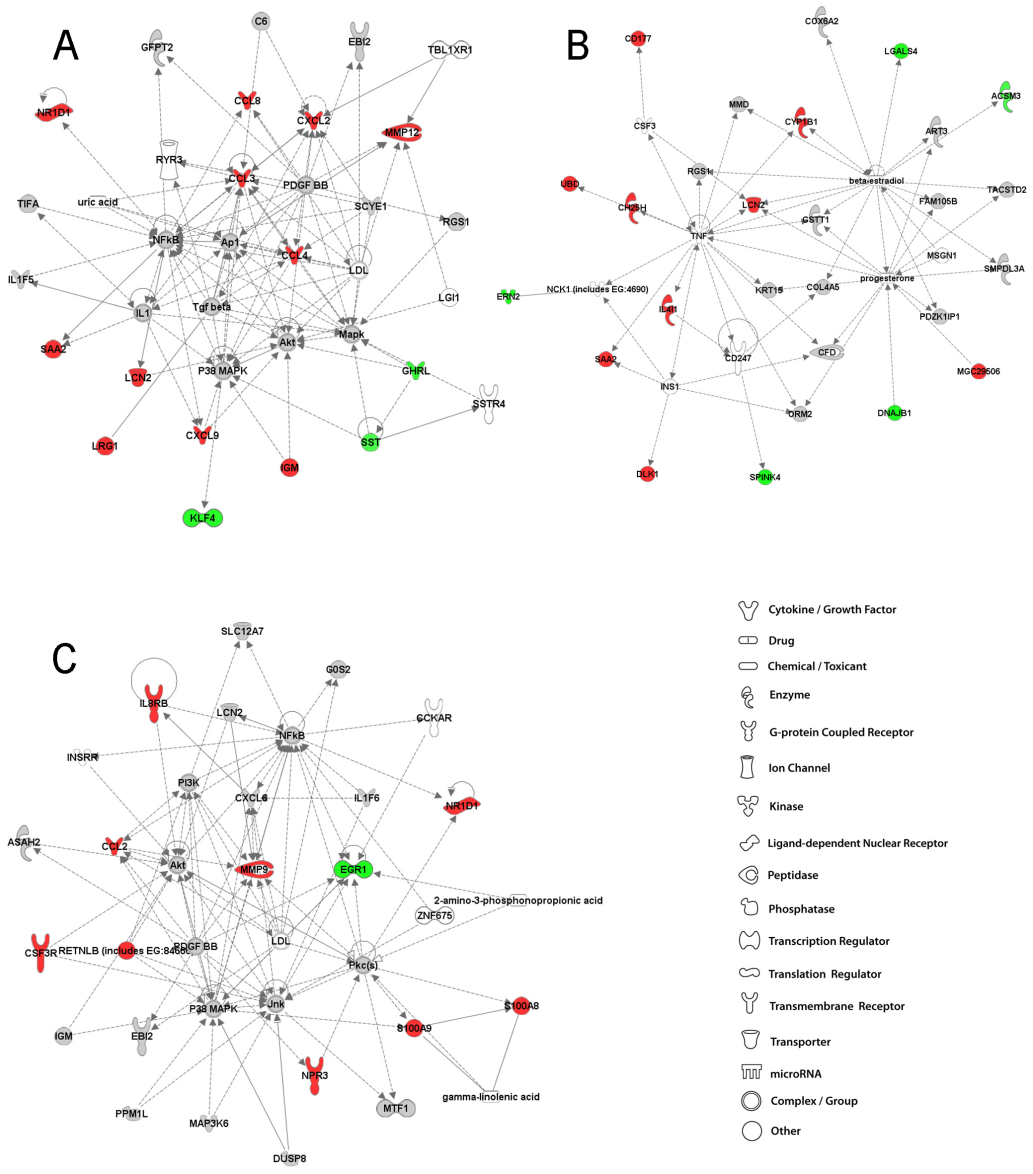
**Top molecular networks 16 weeks after GMA-SS welding fume exposure in A/J and B6 mice**. Gene network analysis by IPA of differentially expressed focus genes 16 weeks after exposure to GMA-SS welding fume in A/J (panel A & B) and B6 (panel C) mice. Whole datasets for each strain were uploaded into IPA then analyzed with the cutoff criteria of ≥1.3 fold change and p < 0.05 versus corresponding sham. Only the highest scoring or most significant network is shown for each group. Intensity of the red (upregulated) or green (downregulated) color indicates level of gene expression. The white color indicates a predicted molecule incorporated from the Ingenuity knowledge base. Gray represents a molecule present in the dataset, but one that did not meet the specified cutoff criteria.

In one of the top two A/J networks, immune response, cell morphology, hematological system development and function, primarily consisted of upregulated genes which were chemokines *CCL3*, *CCL4*, *CCL8*, *CXCL2*, and *CXCL9 *as well as immunoglobulin M (*IgM*), *LCN2*, *MMP12 *and *SAA2 *(Figure 7, panel A). Transcription of macrophage metalloelastase or *MMP12 *was an important and sustained response to both GMA-MS and -SS welding fume in the A/J strain. In contrast, increased *MMP12 *was evident only in response to GMA-SS welding fume at 4 weeks in the B6 lung, but was the top upregulated gene (1.8 fold). Expression levels of this proteolytic gene are primarily associated with macrophages; therefore, its overexpression may reflect an ongoing macrophage accumulation and/or activation in the A/J lung. Further, *MMP12 *was recently shown to play a key role in welding fume-induced lung inflammation as well as in fibrotic diseases such as asbestosis [12,26,27]. Nine genes in the other top network, drug metabolism, lipid metabolism and small molecule biochemistry were upregulated including cytochrome P450, family 1, subfamily B, polypeptide 1 (*CYP1B1*), ubiquitin D (*UBD*), cholesterol 25-hydroxylase (*CH25H*), delta-like 1 homolog [Drosophila] (*DLK1*), and interleukin 4 induced 1 (*IL41*) (Figure 7, panel B). These genes all had indirect connectivity to the main predicted gene hub in this network, tumor necrosis factor [TNF superfamily, member 2] (*TNF*).

At 16 weeks, in the B6 GMA-SS-exposed lung the top network was associated with a similar network of genes as 4 weeks post-exposure although the transcriptional activation switched from decreased to increased (Figure 7, panel C). The genes *CSF3R*, *MMP9*, *S100A8, S100A9 *and resistin like beta (*RETNLB*), downregulated at 4 weeks, were transcriptionally activated at 16 weeks post-exposure to GMA-SS fume. Some of these genes suggest neutrophil (*S100A8 and A9*, interleukin 8 receptor beta [*IL8Rβ]*, *CSF3R*) and macrophage recruitment (*CCL2*) and/or perhaps a tissue remodeling response through activation of *MMP9*. Although, because *MMP9 *is predominantly neutrophil-associated and some evidence suggests it may activate or potentiate IL-8, an inflammatory role in this study cannot be excluded [28]. At 16 weeks, the expression of the reported tumor suppressor gene *EGR1*, remained decreased as was found at 4 weeks post-exposure. Predicted molecules included NFκB, which formed a direct connection with *MMP9*, Akt, PI3K, several members of MAPK and metal-regulatory transcription factor 1 (*MTF1*).

### Summary of network discovery after GMA-SS welding fume exposure

Considering the results of our previous comparative study, the A/J strain was predicted to have a sustained lung transcriptional response to GMA-SS welding fume compared to the B6 [9]. Indeed, this was confirmed by our results, which may further suggest that the A/J strain lacks a necessary anti-inflammatory component to efficiently resolve the lung response to GMA-SS fume. Even at 16 weeks post-exposure, this lung tumor susceptible strain displayed chronic activation of immune response gene networks that included chemokines (*CCL3*, *CCL4*, *CXCL2*, etc) and various immunomodulatory factors such as *LCN2*, *MMP12 *(> 2.5 fold increased at both time points) and *SAA2*. This chronic gene activation to GMA-SS fume supports our long-term histopathological evidence for the presence of perivascular/peribronchial associated lung lymphoid infiltrates--composed of lymphocytes, macrophages, and plasma cells--in the A/J lung at 78 weeks post-exposure and also provides a rationale for further investigation into an enhanced tumorigenic potential of this fume. In contrast, as expected, the B6 exhibited an overall transcriptional downregulation of chemotactic gene signaling, but the later switch to an overexpression for this gene network was surprising. Vast evidence is emerging for the involvement of the leukocyte chemotaxis genes *S100A8 and *S*100A9 *(calgranulins) in inflammation-associated cancer which makes the co-upregulation at 16 weeks in the B6 strain intriguing [29]. The dysregulation of the calgranulins *S100A8 *and *A9*, in addition to *CCL2 *and *IL8Rβ*, warrants further investigation into a possible delayed inflammatory, fibrotic or perhaps proliferative response in this lung tumor resistant strain. Furthermore, involvement of *MMP9 *and possibly of the "cell-survival" Akt signaling pathway in the B6 may represent a generalized lung response to carcinogenic metals. Mechanistic data in the BALB/cJ mouse, an intermediate lung tumor susceptible strain, exposed to repetitive particulate Cr (VI) suggests these genes are important in the lung genotoxic response to this metal [30,31].

### Functional analysis of the lung response after GMA-MS and GMA-SS exposure

Reported in tables 1, 2, 3 and 4 are the associated categories of diseases and disorders, molecular and cellular functions and physiological system development and functions for A/J and B6 mice 4 and 16 weeks post-exposure to GMA-MS and -SS welding fume. The range of associated p-values indicates that each higher level functional category contains more than one lower level functional category. Within each higher level category, the number and regulation of the genes is shown. As presumed, genes often overlapped among the functional categories within a strain. Some functional categories overlapped, but gene subsets were different between the strains as predicted from the network analysis.

**Table 1:** Functional analysis for the lung response 4 weeks post-exposure to GMA-MS welding fume^a^

Strain	Diseases and disorders	p-value^b^	# of genes (up,down)
A/J	Connective tissue disorders	5.92E-07 - 3.04E-02	10 (10,0)
	Immunological disease	5.92E-07 - 3.39E-02	11 (11,0)
	Inflammatory disease	5.92E-07 - 3.11E-02	11 (11,0)
	Skeletal and muscular disorders	5.92E-07 - 3.04 E-02	11 (11,0)
	Organismal injury and abnormalities	6.90E-05 - 8.58E-03	6 (5,1)
B6	Cancer	9.46E-10 - 2.51E-03	14 (3,11)
	Cardiovascular disease	1.13E-08 - 1.25E-03	9 (2,7)
	Connective tissue disorders	3.71E-08 - 2.51E-03	8 (1,7)
	Immunological disease	3.71E-08 - 2.51E-03	9 (1,8)
	Inflammatory disease	3.71E-08 - 1.32E-03	8 (1,7)
			
**Strain**	**Molecular and Cellular Functions**	**p-value^b^**	**# of genes (up,down)**

A/J	Cellular movement	2.41E-05 - 3.28E-02	14 (11,3)
	Cell-to-cell signaling and interaction	5.91E-04 - 3.28E-02	9 (6,3)
	Post-translational modification	6.16E-04 - 1.54E-02	5 (4,1)
	Cellular development	9.42E-04 - 3.39E-02	7 (5,2)
	Cell death	1.24E-03 - 3.39E-02	13 (9,4)
B6	Cell death	9.46E-10 - 2.60E-03	14 (3,11)
	Cellular movement	4.44E-08 - 2.51E-03	11 (2,9)
	Cellular development	5.74E-08 - 2.51E-03	11 (2,9)
	Cellular growth and proliferation	1.52E-07 - 2.51E-03	15 (3,12)
	Cell cycle	1.52E-07 - 2.54E-03	7 (0,7)
			
**Strain**	**Physiological System Development and Function**	**p-value^b^**	**# of genes (up,down)**

A/J	Hematological system development and function	2.41E-05 - 3.37E-02	14 (10,4)
	Immune response	4.67E-05 - 3.22E-02	14 (11,3)
	Behavior	3.57E-04 - 3.95E-04	3 (3,0)
	Nervous system development/function	3.57E-04 - 3.11E-02	7 (7,0)
	Cardiovascular system development/function	2.87E-03 - 3.28E-02	5 (3,2)
B6	Tissue morphology	1.63E-06 - 2.51E-03	9 (0,9)
	Connective tissue development/function	8.69E-06 - 2.51E-03	8 (0,8)
	Skeletal and muscular system development/function	1.50E-05 - 2.57E-03	9 (1,8)
	Cardiovascular system development/function	2.03E-05 - 2.51E-03	7 (2,5)
	Nervous system development/function	2.34E-05 - 2.51E-03	6 (1,5)

^a^Higher level functional categorization and the associated p-values for genes that met the criteria for significance (≥1.3 fold change and p < 0.05). ^b^Range of p-values indicates higher level functions that contained multiple lower level functions. GMA-MS-Gas metal arc-mild steel.

The 4 week analysis revealed genes associated with cancer that functioned primarily in cell death were significant in B6 lung response to GMA-MS welding fume (Table 1). These included, for example, the *FOS *gene family, *KLF2*, *KLF4*, *EGR1 *and *IL1β*, all downregulated, and upregulated genes angiopoietin-related protein 4 (*ANGPTL4*), *CCL6 *and *NR1D1*. By 16 weeks, the majority of these genes were not differentially expressed in the B6 lung and the cancer category then included upregulated chemotactic genes (CCL2, CXCL2) which reflected the cell movement molecular and cellular function (Table 2). Overall, however, the dominant disease and disorder categories in the B6 at both time points were connective tissue disorders, inflammatory disease and immune response which also predominated at 4 weeks in the A/J strain. At 16 weeks in the A/J lung, cancer genes were implicated in the late response to GMA-MS welding fume and cellular compromise was a significant function. In addition, the cell death response (*LCN2*, *DNAJB1*, *ZBTB16*, etc.), perpetuated at this time point and gene number was maintained from 4 weeks (Tables 1 and 2). This may indicate a defective or inadequate apoptotic response in the A/J lung after GMA-MS exposure. In summary, although cancer genes were implicated in both strains to GMA-MS welding fume, their primary role was likely not one of lung tumorigenesis in this experimental scenario.

**Table 2:** Functional analysis for the lung response 16 weeks post-exposure to GMA-MS welding fume^a^

Strain	Diseases and disorders	p-value^b^	# of genes (up,down)
A/J	Cancer	2.14E-04 - 4.71E-02	19 (7,12)
	Gastrointestinal disease	2.14E-04 - 3.72E-02	6 (1,5)
	Renal and urological disease	9.37E-04 - 3.18E-02	4 (1,3)
	Cardiovascular disease	2.69E-03 - 4.73E-02	4 (2,2)
	Connective tissue disorders	2.69E-03 - 5.37E-03	1 (0,1)
B6	Connective tissue disorders	4.66E-04 - 5.37E-03	4 (3,1)
	Immunological disease	4.66E-04 - 4.11E-02	5 (4,1)
	Inflammatory disease	4.66E-04 - 3.08E-02	4 (3,1)
	Skeletal and muscular disorders	4.66E-04 - 2.76E-02	4 (3,1)
	Cancer	4.99E-04 - 3.91E-02	7 (5,2)
			
**Strain**	**Molecular and cellular functions**	**p-value^b^**	**# of genes (up,down)**

A/J	Cellular compromise	1.03E-06 - 1.03E-06	4 (0,4)
	Cellular function and maintenance	9.60E-06 - 3.44E-02	8 (3,5)
	Gene expression	2.12E-05 - 4.98E-02	9 (3,6)
	Cell death	6.95E-05 - 4.73E-02	17 (8,9)
	Cellular development	2.72E-04 - 4.99E-02	13 (6,7)
B6	Cellular movement	7.17E-05 - 4.11E-02	5 (4,1)
	Cell-to-cell signaling and interaction	5.22E-04 - 4.08E-02	5 (5,0)
	Cell morphology	1.08E-03 - 3.60E-02	4 (3,1)
	Cellular assembly and organization	1.08E-03 - 1.92E-02	3 (1,2)
	Cellular development	1.08E-03 - 4.11E-02	7 (5,2)
			
**Strain**	**Physiological System Development and Function**	**p-value^b^**	**# of genes (up,down)**

A/J	Organismal development	2.54E-04 - 4.99E-02	9 (4,5)
	Connective tissue development/function	2.05E-03 - 4.99E-02	7 (1,6)
	Tissue morphology	2.05E-03 - 4.53E-02	10 (4,6)
	Digestive system development/function	2.69E-03 - 4.48E-02	3 (0,3)
	Embryonic development	2.69E-03 - 4.99E-02	6 (3,3)
B6	Behavior	2.09E-07 - 4.89E-05	4 (2,2)
	Nervous system development/function	2.09E-07 - 3.80E-02	7 (5,2)
	Hematological system development/function	7.17E-05 - 3.70E-02	5 (5,0)
	Immune response	7.17E-05 - 4.01E-02	5 (5,0)
	Tumor morphology	4.99E-04 - 2.76E-02	2 (2,0)

^a^Higher level functional categorization and the associated p-values for genes that met the criteria for significance (≥1.3 fold change and p < 0.05). ^b^Range of p-values indicates higher level functions that contained multiple lower level functions. GMA-MS-Gas metal arc-mild steel.

The functional analysis of GMA-SS welding fume exposure confirmed that genes involved in inflammatory disease, immunological disease, connective tissue disorders and skeletal and muscular disorder were significant in both A/J and B6 mice (Table 3 and 4). However, as previously mentioned, the gene networks, numbers and regulation contrasted between the strains. The diseases and disorders that differed between the strains in response to GMA-SS fume at 4 and 16 weeks post-exposure were cancer in the A/J strain and hematological disease in the B6. Hematological disease included leukocytosis, or an increase in white blood cells (primarily neutrophils), as a lower level category. This confirmed the network analysis which showed that genes involved in this process (*MMP9*, *S100A8, S100A*9, etc.) initially downregulated, were then activated in the B6 lung at 16 weeks post-exposure. The interpretation of our finding that there is a switch from a protective, anti-inflammatory, response to a pro-inflammatory response in the B6 lung is difficult, but particle persistence in the lung may play a role.

**Table 3:** Functional analysis for the lung response 4 weeks post-exposure to GMA-SS welding fume^a^

Strain	Diseases and disorders	p-value^b^	# of genes (up,down)
A/J	Connective tissue disorders	3.84E-15 - 5.61E-04	17 (17,0)
	Immunological disease	3.84E-15 - 2.50E-03	20 (20,0)
	Inflammatory disease	3.84E-15 - 2.82E-03	19 (19,0)
	Skeletal and muscular disorders	3.84E-15 - 3.04E-03	19 (18,1)
	Cancer	5.03E-07 - 3.05E-03	24 (20,4)
B6	Inflammatory disease	5.17E-09 - 1.95E-03	12 (3,9)
	Connective tissue disorders	2.08E-08 - 1.95E-03	9 (1,8)
	Skeletal and muscular disorders	2.08E-08 - 1.95E-03	11 (3,8)
	Immunological disease	2.80E-08 - 4.08E-04	11 (2,9)
	Hematological disease	2.76E-07 - 1.49E-03	8 (1,8)
			
**Strain**	**Molecular and cellular functions**	**p-value^b^**	**# of genes (up,down)**

A/J	Cellular movement	2.53E-13 - 2.94E-03	17 (17,0)
	Cell-to-cell signaling and interaction	5.99E-10 - 3.05E-03	17 (16,1)
	Cell morphology	9.53E-09 - 2.82E-03	12 (12,0)
	Cellular assembly and organization	5.30E-07 - 2.33E-04	6 (6,0)
	Cell signaling	7.86E-07 - 2.05E-03	12 (12,0)
B6	Cellular movement	3.03E-09 - 2.04E-03	11 (2,9)
	Cellular development	2.76E-07 - 1.90E-03	13 (4,9)
	Cell-to-cell signaling and interaction	3.41E-07 - 1.49E-03	10 (2,8)
	Cell cycle	3.78E-07 - 2.13E-03	7 (1,6)
	Post-translational modification	8.34E-07 - 1.49E-03	4 (2,2)
			
**Strain**	**Physiological System Development and Function**	**p-value^b^**	**# of genes (up,down)**

A/J	Hematological system development/function	2.53E-13 - 3.04E-03	21 (21,0)
	Immune response	2.53E-13 - 2.28E-03	23 (23,0)
	Immune and lymphatic system development/function	5.99E-10 - 3.04E-03	19 (19,0)
	Tissue development	2.83E-07 - 3.05E-03	11 (10,1)
	Tissue morphology	2.28E-06 - 9.11E-04	10 (10,0)
B6	Hematological system development/function	3.03E-09 - 1.53E-03	11 (2,9)
	Immune response	3.03E-09 - 1.49E-03	10 (2,8)
	Immune and lymphatic system development/function	8.99E-09 - 1.53E-03	10 (2,8)
	Tissue morphology	8.99E-09 - 1.53E-03	10 (2,8)
	Tissue development	1.46E-08 - 1.49E-03	11 (3,8)

^a^Higher level functional categorization and the associated p-values for genes that met the criteria for significance (≥1.3 fold change and p < 0.05). ^b^Range of p-values indicates higher level functions that contained multiple lower level functions. GMA-SS-Gas metal arc-stainless steel.

Cancer persisted at 16 weeks only in the A/J strain and contained a large number of upregulated genes such as *C13ORF15*, *LRG1*, *LCN2*, *MMP12 *and *SAA2 *(Table 4). Furthermore, at 16 weeks cellular growth and proliferation was an important molecular and cellular function that occurred in the A/J lung and differential regulation of genes such as *IL4I*, *NR1D1*, *IGM*, *ZBTB16*, and *KLF4 *were noted. Cell morphology (modification, shape change, and conversion of cells) and effects on cell cycle were also significant gene functions in the A/J lung after GMA-SS fume exposure and it may be of importance that these functions were not represented after GMA-MS fume exposure. Overall, the functional analysis indicates that this welding fume has continued deleterious effects on the lung which may enhance its tumorigenic potential in the A/J strain.

**Table 4:** Functional analysis for the lung response 16 weeks post-exposure to GMA-SS welding fume^a^

Strain	Diseases and disorders	p-value^b^	# of genes (up,down)
A/J	Inflammatory disease	5.57E-06 - 7.31E-03	12 (8,4)
	Connective tissue disorders	2.88E-05 - 5.13E-03	8 (7,1)
	Immunological disease	2.88E-05 - 7.69E-03	10 (9,1)
	Skeletal and muscular disorders	2.88E-05 - 7.69E-03	7 (7,0)
	Cancer	9.61E-05 - 7.69E-03	21 (14,7)
B6	Connective tissue disorders	1.29E-08 - 5.01E-03	8 (7,1)
	Inflammatory disease	1.29E-08 - 5.01E-03	8 (7,1)
	Skeletal and muscular disorders	1.29E-08 - 3.58E-03	7 (7,0)
	Immunological disease	2.26E-08 - 5.01E-03	8 (7,1)
	Hematological disease	8.16E-07 - 3.58E-03	6 (5,1)
			
**Strain**	**Molecular and cellular functions**	**p-value^b^**	**# of genes (up,down)**

A/J	Cell morphology	6.31E-08 - 7.69E-03	9 (7,2)
	Cell cycle	3.91E-06 - 7.59E-03	8 (5,3)
	Cell-to-cell signaling and interaction	6.45E-06 - 7.69E-03	11 (8,3)
	Cellular function and maintenance	6.45E-06 - 5.13E-03	6 (3,3)
	Cellular growth and proliferation	6.45E-06 - 7.69E-03	15 (10,5)
B6	Cellular movement	6.85E-10 - 5.01E-03	8 (7,1)
	Cell-to-cell signaling and interaction	1.37E-08 - 4.52E-03	7 (6,1)
	Cellular growth and proliferation	5.17E-07 - 4.96E-03	9 (7,2)
	Lipid metabolism	1.41E-06 - 3.44E-04	2 (2,0)
	Molecular transport	1.41E-06 - 3.32E-03	4 (4,0)
			
**Strain**	**Physiological System Development and Function**	**p-value^b^**	**# of genes (up,down)**

A/J	Hematological system development/function	6.31E-08 - 7.69E-03	13 (10,3)
	Immune and lymphatic system development/function	6.45E-06 - 7.69E-03	13 (10,3)
	Immune response	1.21E-05 - 7.69E-03	13 (11,2)
	Organismal development	3.08E-05 - 7.50E-03	8 (5,3)
	Embryonic development	3.86E-05 - 5.13E-03	3 (3,0)
B6	Hematological system development/function	2.25E-10 - 5.01E-03	7 (6,1)
	Immune response	2.25E-10 - 5.01E-03	7 (6,1)
	Immune and lymphatic system development/function	2.25E-10 - 4.30E-03	7 (6,1)
	Tissue development	2.25E-10 - 4.30E-03	7 (6,1)
	Tissue morphology	3.09E-08 - 4.30E-03	7 (6,1)

^a^Higher level functional categorization and the associated p-values for genes that met the criteria for significance (≥1.3 fold change and p < 0.05). ^b^Range of p-values indicates higher level functions that contained multiple lower level functions. GMA-SS-Gas metal arc-stainless steel.

### Confirmation of microarray gene expression by RT-qPCR

**Figure 8:**
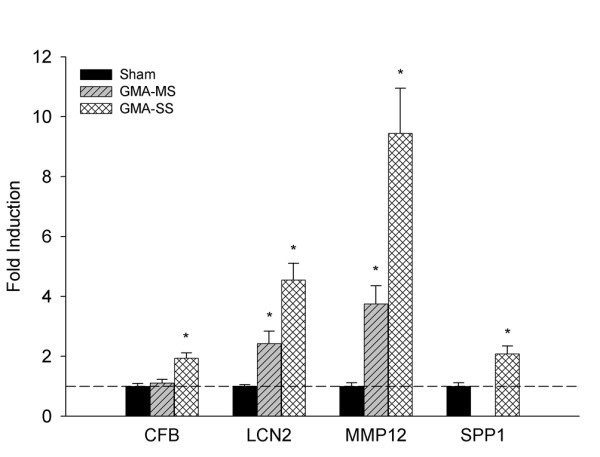
**RT-qPCR confirmation of microarray gene expression changes in welding fume-exposed A/J mice**. Confirmation of microarray gene expression by RT-qPCR for *CFB*, *LCN2*, *MMP12 *and *SPP1 *in whole lung tissue from A/J mice 4 weeks post-exposure to GMA-MS or GMA-SS welding fume (n = 5-6). Data are presented as fold change from sham (dotted line). *-indicates a significant difference from sham (p < 0.05).

Validation of gene expression for *CFB*, *LCN2*, *MMP12 *and *SPP1 *was done by RT-qPCR from 4 week A/J GMA-MS and -SS lung samples (Figure 8). Microarray results showed *CFB *and *SPP1 *were both increased 1.39 fold after GMA-SS exposure only, a value near the selected fold change cutoff for IPA; the significant induction was confirmed by real time RT-PCR supporting the expression value chosen for IPA. Similar corresponding validation was found for *LCN2 *and *MMP12 *(Figure 6). Of note, the real time RT-PCR fold induction values were higher compared to microarray adding further to the utilization of a 1.3 fold cutoff.

## Conclusions

Previously, in A/J and B6 mice, we found strain-dependent differences in terms of the degree and resolution of the lung response, assessed by BAL, to welding fume and a greater toxic potential of carcinogenic metal-containing GMA-SS welding fume [9]. Here, our comprehensive lung transcriptional profiling also revealed significant differences, at the transcriptome level, in the regulation and expression of welding fume-induced gene networks between these mouse strains. In general, lung transcriptional effects were more marked in the susceptible A/J strain, and GMA-SS fume exposure was associated with chronic overexpression of inflammatory genes. This transcriptional response supports our previous finding that GMA-SS is more potent in inducing a chronic immune response in the A/J lung [9]. In pulmonary diseases such as chronic obstructive pulmonary disease, chronic inflammation is considered central to the development of lung cancer. In fact, the link between lung inflammation is not new and evidence exists in several organ systems [32,33]. In the A/J mouse model, anti-inflammatory drugs have been shown to inhibit tumorigenesis, which further suggests inflammation and tumorigenesis are linked [34]. Based on our comprehensive gene profiling, the presence of a chronic inflammatory mileu may allow for the possible genotoxic characteristics of welding fume to be recognized in this susceptible model.

GMA-MS is considered a welding fume of low toxicity compared to the carcinogenic metal-containing SS fumes [11]. Therefore, it was interesting that these fumes were associated with modification of behavioral gene networks in both A/J and B6 mice. This finding highlights novel discoveries gained by using the present methodology. Certainly, further investigation into this behavioral gene subset and its role in welding fume-induced lung toxicity is necessary.

In summary, our results provide unique insight into strain- and welding fume-dependent genetic factors involved in the mouse lung response to welding fume. The gene regulation and network interconnectivity reported in this study reveal possible mechanisms that may differ between lung tumor susceptible and resistant mouse strains exposed to welding fume. Ultimately, this comprehensive analysis will allow us to further, more specifically, probe the complex mechanisms associated with welding fume-induced lung toxicity.

## Competing interests

The authors declare that they have no competing interests.

## Authors' contributions

PCZE performed the animal exposures, isolated the RNA, ran the arrays and the IPA analysis and drafted the manuscript. MLK and SL were responsible for the statistical design, data management, statistical analysis and clustering analysis for these studies. JMA and PCZE conceived and designed the study. All authors read and approved the final manuscript.
